# *Obrieniolus*, a new monotypic genus of Naupactini (Coleoptera, Curculionidae, Entiminae) from the Peruvian Andes and its phylogenetic placement

**DOI:** 10.3897/zookeys.102.1240

**Published:** 2011-06-02

**Authors:** M. Guadalupe del Río, Analía A. Lanteri

**Affiliations:** División Entomología, Museo de La Plata, Paseo del Bosque s/n, 1900 La Plata, Argentina

**Keywords:** *Obrieniolus robustus*, new taxa, phylogeny, Paramo-Puna subregion, South American Transition Zone

## Abstract

A new monotypic genus of *Naupactini* (*Coleoptera*: *Curculionidae*), *Obrieniolus* del Río is described based on the new species *Obrieniolus robustus* del Río, endemic to Peru. This genus is easily recognized by the black, denuded and shiny integument, with imbricate microsculpture and the rounded body, with short, cordiform and moderately convex elytra. According to a cladistic analysis based on 69 continuous and discrete morphological characters, the new genus is the sister taxon of a group formed by *Amitrus* Schoenherr, *Trichocyphus* Heller, *Amphideritus* Schoenherr, *Asymmathetes* Wibmer & O’Brien and *Galapaganus* Lanteri. The paper includes habitus photographs, line drawings of genitalia, mouthparts, and other external features of taxonomic value, and a dichotomous key to the genera of *Naupactini* distributed in the South American Transition Zone.

## Introduction

The tribe *Naupactini* (*Curculionidae*: *Entiminae*) consists of approximately 65 genera (Alonso-Zarazaga and Lyal 1999) with over 500 species mainly distributed in Central and South America (Wibmer and O´Brien 1986). Six genera and 28 species of this tribe have been reported for the Paramo-Puna subregion of the Andean region (Cabrera and Willink 1973; Morrone 2001) or Central-Northern area of the South American Transition Zone *sensu* Morrone (2006). These genera are *Amitrus* Schoenherr, 1840 (8 spp.), *Amphideritus* Schoenherr, 1840 (8 spp.), *Asymmathetes* Wibmer & O’Brien, 1986 (7 spp.), *Galapaganus* Lanteri, 1992 (15 spp., only two in this region), *Melanocyphus* Jekel, 1875 (2 spp.), and *Trichocyphus* Heller, 1921 (1 sp.). Some of them have been revised (Lanteri 1989, 1992; del Río and Lanteri 2007) and the remaining are currently under revision (del Río 2010). Their species diversity is poorly known, the phylogenetic relationships among them have never been assessed, and there is scarce information on host plants and biological aspects, even though some species are possible potato pests (Munro 1968; Peña 2001).

In the present contribution we describe a new Andean genus and species which cannot be accommodated within any of the existing weevil genera. This new monotypic taxon is endemic to Peru and ranges throughout the Puna province, mainly characterized by a shrublike steppe, with bushes 40 to 150 cm high. A cladistic analysis was performed to analyze the relationship of the new genus with other *Naupactini* from the Andes and the Pacific coastal deserts, a monophyletic clade within this tribe (del Río and Lanteri unpublished).

## Materials and methods

The material studied comes only from the Charles W. O´Brien personal collection (CWOB). The holotype and three paratypes have been returned to CWOB collection, and one paratype has been deposited in the Museo de La Plata collection (MLP).

Dissections of female and male genitalia were done according to standard entomological techniques. Measurements were taken with an ocular micrometer. Abbreviations used in the description are as follows: LB: length of body, measured from apex of rostrum to apex of elytra; WRa: width of rostrum across apex; WRb: width of rostrum at base; LR: length of rostrum from anterior margin of eye to apex; LA: maximum length of antenna; A1: length of funicular article 1; A2: length of funicular article 2; WC: maximum width of club; LC: maximum length of club; WP: maximum width of pronotum; LP: maximum length of pronotum; WE: maximum width of elytra; LE: maximum length of elytra. For line drawings we used a camera lucida adapted to a stereoscopic microscope Nikon MZ1000.

**Phylogenetic analysis.** The data matrix (see Appendix 1) includes 13 terminal species of *Naupactini*, belonging to eight genera: *Amitrus* (*Amitrus alutaceus* and *Amitrus mundus*); *Amphideritus* (*Amphideritus vilis* and *Amphideritus puberulus*); *Asymmathetes* (*Asymmathetes pascoei* and *Asymmathetes nigrans*); *Galapaganus* (*Galapaganus femoratus* and *Galapaganus galapagoensis*); *Melanocyphus* (*Melanocyphus bispinus* and *Melanocyphus lugubris*); *Trichocyphus* (*Trichocyphus formosus*); *Mendozella* (*Mendozella curvispinis*); and the new genus *Obrieniolus* (*Obrieniolus robustus*). Each genus is represented by two species (one of them the type species), except for those that are monotypic.

The 69 characters selected (table 1) correspond to the external morphology (54) and to the genitalia (10 of females and five of males). Sixteen continuous characters correspond to ranges of ratios between measurements and were treated as such, avoiding the use of *ad hoc* methods to establish ranges (Goloboff et al. 2008). Multistate characters with intraspecific variation were treated as polymorphic, as indicated in TNT (e.g. [0 1]). All discrete characters were treated as unordered.

Parsimony analysis was performed with the software “Tree Analysis using New Technologies” (TNT) (Goloboff et al. 2003) using the ‘traditional’ search approach based on 100 replicates using TBR branch swapping, and hold 10. Discrete characters were mapped on the most parsymonious cladogram through Winclada version 1.00.08 (Nixon 2002). Homoplasy was estimated using consistency and retention indices (Kluge and Farris 1969; Farris 1989). Branch support was evaluated by bootstrap (Felsenstein 1985) with 100 replicates, and values over 50% were indicated below each branch (Fig. 14).

The most parsimonious tree was rooted with *Mendozella curvispinis*, which is the only terminal taxon distributed in the Monte province, belonging to the South American Transition Zone but not to the Paramo-Puna subregion (Lanteri 1989; Lanteri and Morrone 1991).

## Taxonomy

### Obrieniolus

del Río gen. n.

urn:lsid:zoobank.org:act:DF6EDE47-07C1-4E1B-A2CE-6D01D6970930

http://species-id.net/wiki/Obrieniolus

#### Type species.

New species *Obrieniolus robustus* del Río.

#### Diagnostic description.

**Figures 1–3.**
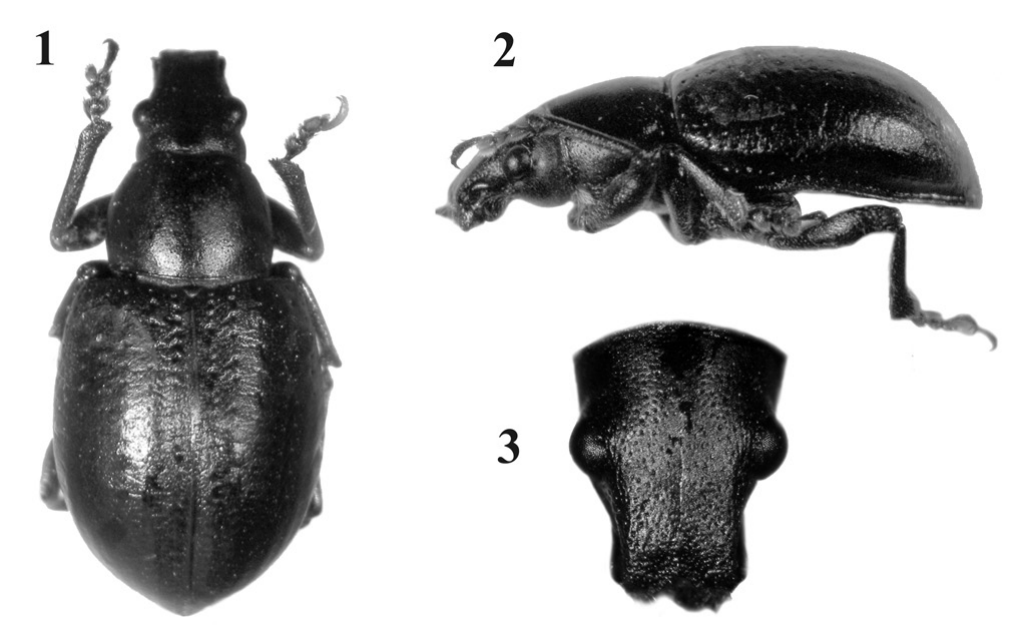
*Obrieniolus robustus* **sp. n.**, holotype **1** habitus, dorsal **2** habitus, lateral **3** head and rostrum, dorsal.

**Figures 4–7.**
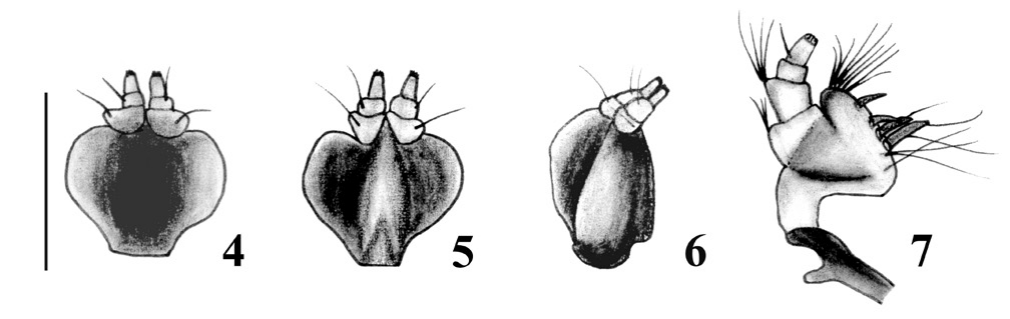
Mouthparts of *Obrieniolus robustus* **sp. n.**. **4** prementum, external view **5** prementum, internal view **6** prementum, lateral view **7** left maxilla.

Body rounded and medium-sized; integument black, denuded and shiny, with imbricate microsculpture and reddish-brown tarsi (Figs 1–2); rostrum very short with narrow epistome (Fig. 3); maxillae with suboval mala, not excavate, almost parallel to longitudinal axis of palpus (Fig. 7); prementum subcordate without setae (Figs 4–6); posterior margin of pronotum constricted and slightly posteriorly “V” shaped; elytra cordiform, moderately convex, with slightly posteriorly curved base and slightly prominent and subquadrate humeri (Fig. 1); punctures of striae strongly separated from each other; scutellum tiny, denuded; front coxae slightly separated from each other, 3× closer to anterior than to posterior margin of prosternum; row of denticles only present in front tibiae; outer bevels of hind tibiae broad and oblique. Ovipositor thin and curved in lateral view, longer than abdomen (Fig. 11); sternite VIII with subrhomboidal elongate plate and apodeme *ca.* 2× longer than plate (Fig. 10); spermathecal duct very long, membranous and sinuous (Fig. 13).

#### Etymology.

The genus is named after the outstanding weevil specialist Charles W. O´Brien, who loaned us the material for this study.

#### Remarks.

*Obrieniolus* is distinguished by the particular shape of the body (cordiform, extremely rounded and short), completely covered with imbricate microsculpture, the strongly separated punctures of the elytral striae, and the bursa copulatrix studded with dense and minute spines directed backwards, near the vagina. Other generic characters are common in most *Naupactini* inhabiting mountain environments, **e.g.** the black, denuded and strongly sclerotized integument, the absence of metathoracic wings and the reduced shoulders.

#### Natural history.

*Obrieniolus* seems to be endemic to northeastern Peru, Department of La Libertad, at about 2800 m of elevation. Its distribution corresponds to the Puna biogeographic province, that also extends in eastern Bolivia, northern Argentina and Chile (Morrone 2006), which is a steppe shrublike formation with bushes 40 to 150 cm high. The area where *Obrieniolus* occur is close to the Coastal Peruvian Desert province, a narrow strip along the Pacific coast from northern Peru to northern Chile (Morrone 2006), characterized by the extremely dry climate.

*Obrieniolus robustus* was found under rocks, in dry hills with grasses and sparse small shrubs. No specific host plant associations are known. The possibility of parthenogenesis is inferred based on the absence of males. This kind of reproduction seems to be frequent in the Andean species of *Naupactini* (Lanteri and Normark 1995; del Río 2010).

#### Obrieniolus robustus

del Río sp. n.

urn:lsid:zoobank.org:act:3DDE1999-9F5C-4698-B296-9B06BE9A6604

http://species-id.net/wiki/Obrieniolus_robustus

##### Etymology.

The species epithet is an adjective alluding to one of the distinctive characters of the new species, which is its robust body shape.

##### Material examined.

**Holotype.** Female, 10.8 mm long, with labels as follows “Perú, La Libertad Dept.,Otuzco P., 1.2 mi NW Agallpampa, on rd. to Trujillo, 2840m, under rocks on dry hillside with sparse brown grass, sparse small brown plants, XI-27-1977, G. Noonan & M. Moffett”. Pinned with genitalia dissected and placed in a microvial with glycerin. Deposited at CWOB.

##### Type locality.

Perú: Department of La Libertad,Otuzco, Agallpampa.

##### Paratypes.

4 ♀♀ same data as holotype (3 CWOB; 1 MLP).

##### Description.

**Figures 8–13.**
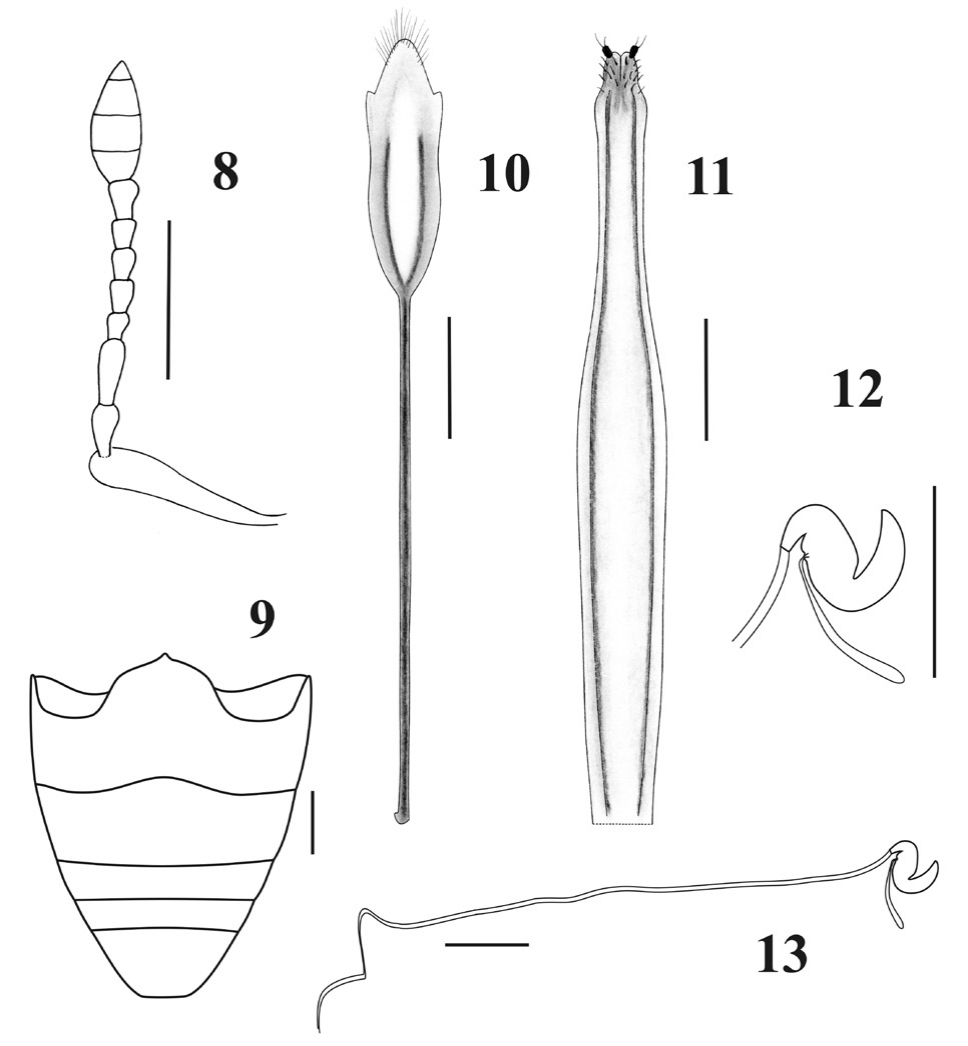
Antennae, ventrites and female genitalia of *Obrieniolus robustus* **sp. n.** **8** left antenna **9** ventrites **10** sternite VIII **11** ovipositor, ventral view **12** spermatheca **13** spermatheca with spermathecal duct. Scale line: 1 mm.

*Female.*Species medium sized (LB: 8.5–10.8 mm), body broadly rounded (Figs 1–2). *Integument* visible, dark, with imbricate microsculpture, tarsi reddish brown. *Vestiture*. Dorsum naked of scales; pleura (mesepimeron and metaepisternum) covered with whitish setiform scales; legs and venter with disperse short yellowish decumbent setae, longer and more abundant in inner face of femur and tibia. *Rostrum* (Fig. 3) slightly shorter than wide (LR/WRa: 0.87–0.94), sides moderately convergent toward apex (WRb/WRa: 1.39–1.40), dorsum flat, punctate-foveolate (with disperse broad punctures and small punctuation between them); dorso-lateral carinae absent; median groove narrow, extended up to posterior margin of eyes or slightly exceeding them. Epistome slightly depressed, narrow, subtriangular, denudate and with strigose sculpture. Scrobes curved, deep, ending below eyes. *Mouthparts.* Mandibles naked of scales; outer face foveolate, with coarse setae. Maxillae (Fig. 7) with suboval mala, not excavate, almost parallel to longitudinal axis of palpus; basal area with thin long setae (~5), lacinial teeth short, curved and wide (1+3); distal area with wide long setae (~13); palpifer and articles 1–2 of palpi transverse, and article 3 subcylindrical. Prementum (Figs 4–6) subcordate; external surface alveolate, moderately concave and naked of setae; inner surface without setae, with prominent median keel. Palpi smooth, (setae 4-1-0), forming a very open angle with prementum axis. Gular angle near 90° in lateral view. Eyes medium- sized and moderately convex. Preocular depression absent. Frons wide (*ca.* 3x diameter of eye), slightly convex, punctate-foveolate. Vertex slightly convex. Postocular constriction distinct. *Antennae* (Fig. 8) short and robust (LB/LA: 2.80–2.93), covered with wide decumbent setae. Scape slender, reaching middle of eye. Funicular article 2 about 1.2× as long as article 1; funicular articles 3–7 slightly longer than wide (1.5×). Club oval (LC/WC: 2-42-2.47), acuminate.

*Pronotum* (Figs 1–2) subcylindrical, moderately transverse (WR/LR: 1.27–1.32); flanks moderately curved; disc slightly convex, punctate-foveolate, with imbricate microsculpture; median groove absent; anterior margin slightly emarginated, strongly thickened; base posteriorly “V” shaped. S*cutellum* subtriangular, minute, convex, denuded.

*Elytra* (Figs 1–2) subcordate, short (LE/WE: 1.19–1.27), moderately convex, with imbricate microsculpture and finely transversally rugose in the posterior half; base slightly posteriorly curved; humeri subquadrate, slightly prominent; striae well defined, punctures very distant from each other, deep, medium sized in anterior third, smaller in median third and inconspicuous in posterior third; striae 9–10 closer on posterior two-thirds; intervals flat, 3–4× as wide as striae; apical declivity moderately abrupt; apex subacute. Metathoracic wings absent.

*Legs*. Black, naked of scales, with imbricate microsculpture. Front coxae slightly separate, 3x closer to anterior margin than to posterior margin of prosternum (almost reaching anterior margin); protibiae with row of 7–11 acute medium sized denticles and strongly acute mucro; meso and metatibiae without denticles and mucro; metatibial apex with broad outer bevel (placed in whole tibial apex), oblique regarding tibial axis, with small whitish iridescent scales; dorsal comb slightly shorter than apical comb or subequal.

*Abdomen* (Fig. 9) Intercoxal portion of ventrite 1 broader than cavities of hind coxae (1.6–1.7×); ventrite 2 longer than ventrites 3+4 (1.4×); apex of ventrite 5 blunt, slightly emarginated.

*Female genitalia.* Sternite VIII (Fig. 10) with plate subrhomboidal, elongate, having apical tuft of long setae and a pair of lateral sclerotized stripes reaching 2/3 of plate; apodeme 1.8–2× longer than plate. Ovipositor (Fig. 11) slender, very long, curved in lateral view, 1.3–1.35× longer than ventrites 1–5; ventral baculi slender, subparallel; coxites slightly sclerotized; styli well developed, thin, directed backwards. Bursa copulatrix studded with dense and minute spines directed backwards, near the vagina. Spermathecal body (Fig. 12) subcylindrical, strongly sclerotized; nodulus truncate-conical, short; ramus indistinct; cornu of medium length. Spermathecal duct (Fig. 13) very long (~8mm, longer than abdomen) membranous and sinuous.

*Morphometrics*. Holotype, female: rostrum LR/WRa: 0.94, WRb/WRa: 1.4; antenna LB/LA: 2.93, A2/A1:1.17, club LC/WC: 2.42; pronotum WP/LP: 1.27; elytra LE/WE: 1.22; LE/LP: 2.55.

*Male.* Unknown.

## Cladistic analysis: results and discussion

**Figure 14.**
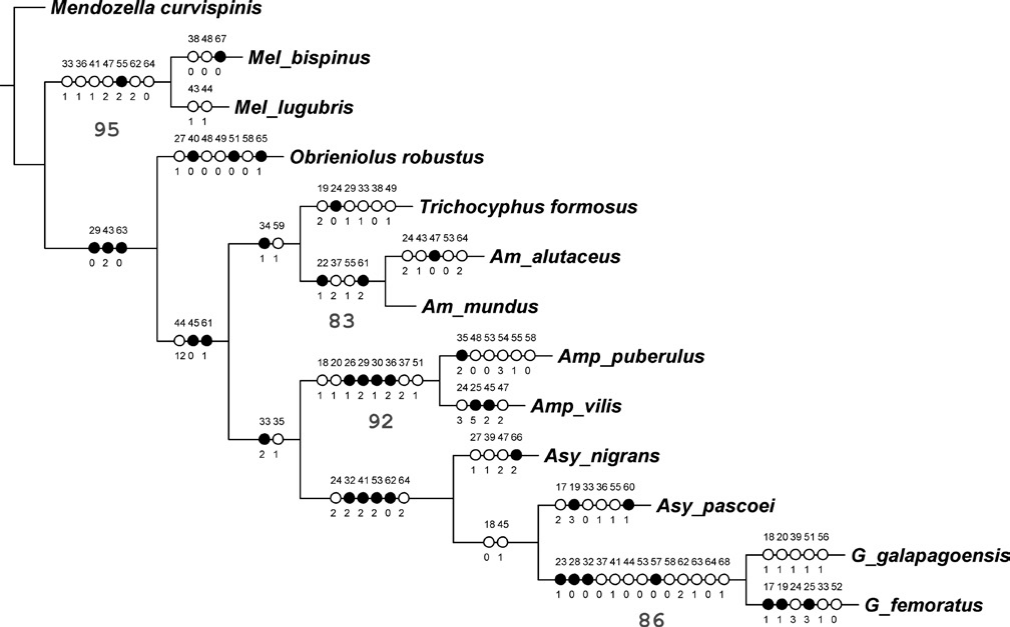
Most parsimonious tree, based on morphological characters, analyzed under equal weights (L 195.20, CI 0.58, RI 0.53). Bootstrap values over 50% below the corresponding branches. Discrete characters (17 to 68) mapped on the branches:open circles=homoplasies, black circles=synapomorphies. Numbers of characters and character states as in table 1.

The parsimony analysis resulted in a single most parsimonious cladogram 195.20 steps long, with CI = 0.58 and RI = 0.53 (Fig. 14). *Melanocyphus* is the sister taxon of the remaining genera, that form a clade justified by eight synapomorphies, such as the narrow epistome (char. 29.0) and the elytral base straight to slightly curved backwards (char. 43.2). Within this clade, *Obrieniolus* is the sister taxon to the remaining *Naupactini* from the Andes and the Pacific coastal deserts of South America. The new genus is characterized byseveral apomorphies. Some of them are continuous characters related to the shape of elytra (char. 9), length of ventrite 2 regarding 3+4 (char. 11) and length of ovipositor (char. 13). Other apomorphies are the rostral sulcus exceeding posterior margin of eyes (char. 27.1), the scutellum indistinct (char. 40.0), the apical declivity of elytra moderately abrupt (char. 48.0), the elytral intervals markedly wider than striae (char. 49.0), the punctures of striae strongly separated from each other (char. 51.0), the plate of sternite VIII of female subrhomboidal, elongate (char. 58.0), and the presence of spines in the bursa copulatrix (char. 65.1).

The sister clade of *Obrieniolus* is divided into two groups, one including *Trichocyphus* and *Amitrus*, and the other, with *Amphideritus*, *Asymmathetes* and *Galapaganus*. The first group is characterized by the wide intercoxal area of ventrite 1 (char. 10), the very stout antennae (char. 34.1) and the row of setae along the ovipositor, on the external side of baculi (char. 59.1). The second group is mainly supported by the gular angle strongly obtuse (char. 33.2) and the antennal scape reaching to slightly exceeding hind margin of eyes (char. 35.1).

Each genus included in the tree was recovered as monophyletic with high nodal support (BP over 80%), except *Asymmathetes*, which is not monophyletic. On the contrary, the relationships among the Andean genera are weakly supported.

The new genus *Obrieniolus* is superficially similar to *Amitrus*, because both have a strongly sclerotized black integument, devoid of scales and are almost lacking setae, and have a distinct sculpture. However, the current cladistic analysis shows that the new genus is not closely related to *Amitrus* or to any other genus, justifying its treatment as a separate generic taxon. Characters such as the strongly sclerotized integument, dull coloured, sculpturate, and usually devoid of scales, as well as the reduction of elytral humeri and metathoracic wings, are common in several groups inhabiting the high Andes, under similar extreme environments.

The Andean *Naupactini* are distributed in different biogeographic provinces of the Paramo-Puna subregion: *Melanocyphus* inhabit the Colombian Paramos; *Obrieniolus* occur in the Northern Puna, in the boundaries of the Peruvian Coastal Desert; *Trichocyphus* and *Amitrus* also inhabit in the Puna, but they reach a southern and broader distribution range; *Amphideritus* have representatives in the Paramos of Venezuela and Colombia, and along the Pacific coastal deserts of Peru and Chile; *Asymmathetes* inhabit the Paramos of Ecuador; and the species of *Galapaganus* inhabit in the Peruvian Coastal Desert, the Galapagos islands and continental Ecuador.

## Key to genera of *Naupactini* from South

**Table:** 

1	Antennae squamose	*Mendozella*
–	Antennae setose	2
2	Cavities of front coxae separated	*Asymmathetes*
–	Cavities of front coxae confluent	3
3	Elytral intervals strongly convex; base of pronotum bisinuate	*Melanocyphus*
–	Elytral intervals moderately convex to flat; base of pronotum not bisinuate	4
4	Mandibular cusp prominent; antennae long, with funicular articles 3–7 markedly longer than wide; front femora much wider than posterior femora; spermathecal body subglobose	*Galapaganus*
–	Mandibular cusp slightly prominent or reduced; antennae medium length to short, with funicular articles 3–7 slightly longer than wide to moniliform; front femora slightly wider than posterior femora to subequal; spermathecal body subcylindrical	5
5	Antennae stout (maximum width of funicular articles about 1\3 high of eye); intercoxal area of ventrite 1 more than 2 times width of cavity of hind coxae	6
–	Antennae moderately stout to slender (maximum width of funicular articles less than ? high of eye); intercoxal area of ventrite 1 less than 2 times width of cavity of hind coxae	7
6	Elytral setae long and erect on entire elytral surface; all pairs of tibiae with row of denticles on inner margin	*Trichocyphus*
–	Elytral setae present only on elytral apex; pro and mesotibia with row of denticles on inner margin, metatibia always lacking denticles	*Amitrus*
7	Elytral setae dense; sides of rostrum strongly curved; epistome elevated and with a transversal callosity separating it from rostrum; pre-epistome reduced to absent; antennal scape curved; elytra suboval	*Amphideritus*
–	Elytral setae absent; sides of rostrum slightly curved; epistome depressed, without transversal callosity; pre-epistome well developed; antennal scape straight to slightly curved; elytra subcordate	*Obrieniolus*

**Table 1.** List of characters, character states and codes

Continuous characters	
0.	Body length in mm, taken from apex of rostrum to apex of elytra (LB).
1.	Ratio between length of rostrum and width of rostrum at apex (LR/WRa).
2.	Ratio between width of rostrum at base and width at apex (WRb/WRa).
3.	Ratio between width of frons and high of eye (WF/He).
4.	Ratio between body length and length of antenna (LB/LA).
5.	Ratio between length of funicular article 2 and 1 (A2/A1).
6.	Ratio between length and width of antennal club (LC/WC).
7.	Ratio between maximum width and length of pronotum (WP/LP).
8.	Ratio between maximum length of elytra and maximum length of pronotum (LE/LP).
9.	Ratio between maximum length and width of elytra (LE/WE).
10.	Ratio between width of intercoxal area of ventrite 1 and width of cavity of hind coxa (Wzi/Wcm).
11.	Ratio between length of ventrite 2 and ventrites 3+4 (L2/L3+4).
12.	Ratio between length of apodeme and plate of sternite VIII (LAE/LPE).
13.	Ratio between length of ovipositor and length of ventrites 1-5 (LOv/Lv).
14.	Ratio between length of aedeagus and length of ventrites 1-5 (LAe/Lv).
15.	Ratio between length of aedeagal apodemes and length of median lobe (LAp/Lml).
Discrete charactersExternal morphology	
16.	Scaly vestiture of antennae: present (0); absent (1).
17.	Scaly vestiture of pronotum: absent (0); scarce (1); abundant (2).
18.	Elytral vestiture: squamose (0); setose (1); scarce or absent (2).
19.	Scaly vestiture of elytra: absent (0); mostly absent, restricted to some areas (1); present in all surface, but not entirely covering the integument (2); present in all surface, completely covering the integument (3).
20.	Elytral vestiture of decumbent setae: absent (0); present, dense (1).
21.	Elytral setae: absent (0); short, suberect (1); long, erect (2).
22.	Setae of the elytral apex: absent (0); present, usually forming a tuft (1).
23.	Scutellum: squamose (0); setose (1); denuded (2).
24.	Rostrum and frons: smooth (0); punctuate or foveolate (1); foveolate-strigose (2); coarsely strigose (3); lacunose (4).
25.	Pronotum: smooth (0); punctuate or foveolate (1); strigose (2); tuberculate (3); coarsely lacunose (4); foveolate-granulose (5).
26.	Sides of rostrum: straight to slightly curved (0); strongly curved (1).
27.	Rostral sulcus: reaching frons (0); exceeding posterior margin of eyes (1).
28.	Rostral carinae: present, strong (0); present, slight (1); absent (2).
29.	Size of epistome: narrow (0); moderately wide (1); very wide (2).
30.	Epistome: depressed (0); elevated, with a posterior transversal callosity (1).
31.	Pre-epistome: absent or reduced (0); well developed (1).
32.	Support of mandibular cusp: prominent (0); slightly projected (1); reduced (2).
33.	Gular angle: about 90° (0); moderately obtuse (1); strongly obtuse (2).
34.	Antennae: slender to moderately stout (0); very stout (1).
35.	Length of antennal scape: short, not reaching hind margin of eyes (0); medium sized, reaching to slightly exceeding hind margin of eyes (1); long, largely exceeding hind margin of eyes (2).
36.	Antennal scape: straight (0); slightly curved (1); moderately curved (2).
37.	Funicular articles 3-7: distinctly longer than wide (0); slightly longer than wide (1); moniliform (2).
38.	Sides of pronotum: almost straight (0); slightly to moderately curved (1); strongly curved (2).
39.	Pronotal base: straight (0); “V” shaped (1); bisinuate (2).
40.	Scutellum: indistinct (0); small to medium sized (1).
41.	Maximum width of elytra: about middle (0); on posterior third (1); on anterior third (2).
42.	Elytral disc: strongly to moderately convex (0); slightly convex to flat (1).
43.	Elytral base: strongly to moderately bisinuate (0); slightly bisinuate (1); straight to slightly curved backwards (2) strongly curved backwards (3).
44.	Elytral humeri: strongly prominent (0); moderately prominent (1); slightly prominent (2).
45.	Humeri: rounded (0); subquadrate (1); oblique (2).
46.	Humeral teeth: absent (0); present, prominent (1).
47.	Elytral apex: rounded (0); subacute (1); acute (2).
48.	Apical declivity of elytra: strongly to moderately abrupt (0); slightly abrupt (1); soft (2).
49.	Elytral intervals: markedly wider than striae (more than 3x) (0); slightly wider than striae (1,5-2x) (1); about same width of striae or slightly slender (2).
50.	Elytral intervals: flat to slightly convex (0); moderately convex (1); strongly convex (2).
51.	Punctures of elytra: strongly separated from each other (0); close to each other (1); very close to each other (2).
52.	Metathoracic wings: present, well developed (0); absent (1).
53.	Front coxae: contiguous (0); slightly separate (1); distinctly separate from each other (2).
54.	Row of denticles on inner margin of tibiae: present on three pairs of tibiae (0); present on front and middle tibiae (1); present only on front tibiae (2); absent on three pairs of tibiae (3).
55.	Outer bevel of metatibial apex: broad, squamose (0); moderately broad, squamose (1); absent (2).
56.	Apical comb of hind tibiae longer than dorsal comb (0); about as long as dorsal comb (1); shorter than dorsal comb (2).
57.	Front femora: more robust than middle and posterior femora (0); subequal (1).
Female genitalia	
58.	Plate of sternite VIII of female: subrhomboidal, elongate (basal half longer than apical half) (0); subrhomboidal, not elongate (basal and apical half subequal) (1).
59.	Rows of setae along sides of baculi (ovipositor): absent (0); present (1).
60.	Coxites: slightly sclerotized (0); moderately to strongly sclerotized, not projecting (1); strongly sclerotized, tapering into a nail-like process and covering styli (2).
61.	Length of spermathecal duct: longer than ovipositor (=long) (0); as long as ½ ovipositor (=medium-sized) (1); shorter than 1/2 ovipositor (=short) (2).
62.	Spermathecal body: subcylindrical, long (0); subcylindrical, short (1); globose (2)
63.	Ramus of spermatheca: indistinct to slightly developed (0); well-developed (1).
64.	Cornu of spermatheca: short (0); medium length to long (1); very long (2).
65.	Spines on bursa copulatrix: absent (0); present (1).
Male genitalia	
66.	Angle between median lobe and its apodemes: almost flat (0); obtuse (1); about 90° (2).
67.	Apex of median lobe: acute (0); subacute (1); rounded, with a pointed projection at apex (2).
68.	Setae on apex of median lobe: absent (0); present (1).

## Supplementary Material

XML Treatment for Obrieniolus

## Appendix I

A data matrix including 13 terminal species of eight genera of Naupactini from the South American Transition Zone.

### Note:

The data matrix with the 13 species of Naupactini can be found on the *ZooKeys* website as a Microsoft Excel file (.xls), doi: 10.3897/zookeys.102.1240.app).
